# Treatment Utilisation and Satisfaction With Management in Individuals With Osteoarthritis and Metabolic Multimorbidity: A Cross‐Sectional Multi‐Country Study

**DOI:** 10.1002/msc.70058

**Published:** 2025-01-23

**Authors:** Filippo Recenti, Andrea Dell'isola, Benedetto Giardulli, Marco Testa, Polina Pchelnikova, Mwidimi Ndosi, Simone Battista

**Affiliations:** ^1^ Department of Neuroscience, Rehabilitation, Ophthalmology, Genetics, Maternal and Child Health University of Genova Genoa Italy; ^2^ Department of Clinical Sciences Clinical Epidemiology Unit Orthopaedics Lund University Lund Sweden; ^3^ Patient Research Partner Moscow Russian Federation; ^4^ School of Health and Social Wellbeing University of the West of England Bristol UK; ^5^ School of Health and Society Centre for Human Movement and Rehabilitation University of Salford Salford UK

**Keywords:** comorbidity, consumer experience, diabetes, healthcare consumption, hypertension, obesity, osteoarthritis

## Abstract

**Purpose:**

To compare treatment utilisation for osteoarthritis (OA) and satisfaction with OA management between individuals with and without comorbid metabolic conditions (e.g., diabetes, obesity, dyslipidaemia, hypertension).

**Methods:**

Secondary analysis of a cross‐sectional international survey study (Italy, Russia, Sweden) on people ≥ 40 years old with knee/hip OA. Metabolic comorbidity was self‐reported. We used direct standardisation with prevalence ratios and mixed‐effect models to estimate the associations between comorbidity with treatment utilisation and satisfaction (score 0–100).

**Results:**

We analysed 401 individuals (48% Sweden, 28% Italy, 24% Russia; 53% with ≥ 1 metabolic condition). Those with and without comorbid metabolic conditions showed similar prevalence for first‐line interventions (exercise, education, and weight management). Metabolically unhealthy individuals showed higher use of opioids (prevalence ratio [95% CI] 1.9 [1.3–2.4]), antidepressants (1.8 [1.1–2.5]), corticosteroid injections (1.4 [1.0−1.8]), and homoeopathic products (2.1 [1.2–3.0]). Satisfaction with care (adjusted difference: −3.9 [95% CI: −8.5 to 2.4]) and information received about treatments (−4.0 [−9.7 to 1.7]) were similar.

**Conclusions:**

While first‐line OA interventions were similarly used, those with metabolic conditions relied more on second‐line and non‐recommended treatments, showing comparable satisfaction. More effort is needed to increase the adoption of lifestyle‐focused treatments in OA and to minimise the use of less recommended options among individuals with metabolic comorbidities.

## 1 Introduction

Osteoarthritis (OA) and metabolic conditions, such as hypertension, type II diabetes, obesity, and dyslipidaemia (Saklayen 2018), are among the top causes of disability worldwide and often co‐exist (Zhuo et al. 2012). Their steadily increasing prevalence is driven mainly by shared common risk factors, such as increased sedentary behaviour and heightened dietary energy intake, resulting in weight gain and systemic low‐grade inflammation (Furman et al. 2019). Moreover, there is growing evidence regarding the existence of a distinctive metabolic OA phenotype (Dell’Isola et al. 2016), where the combination of OA and metabolic conditions can lead to a cycle of pain, physical disability, weight gain, and muscle weakness.

Providing care for individuals with multimorbidity, such as individuals with OA and metabolic conditions, presents important challenges for healthcare providers and service users. OA is often wrongly perceived as a physiological ageing process and could be mistreated or undertreated when other disabling conditions are present (Basedow and Esterman 2015; Hagen et al. 2016; Hunt et al. 2012; Oomen et al. 2022; Theis, Brady, and Sacks 2019). Most healthcare systems are designed to treat single conditions (Skou et al. 2022) and are not prepared to face the complexity of shared decision‐making and prioritisation arising from the coexistence of multiple conditions (Kuipers, Nieboer, and Cramm 2021; Schuttner et al. 2022). This complexity may lead both healthcare professionals and patients to favour passive interventions over active ones, such as exercise and education, which, despite being recommended as first‐line interventions for both OA and metabolic diseases, are often perceived as more time‐consuming and demanding (Teo et al. 2020).

In addition, individuals living with OA and metabolic conditions require more regular and frequent clinical encounters involving different healthcare professionals, often at different facilities, which can result in inconvenient, inefficient, and unsatisfactory management (Bamm, Rosenbaum, and Wilkins 2013; Salisbury 2013). The increased burden of care associated with multimorbidity can negatively impact the satisfaction with OA management. On the other hand, it can be interpreted as enhanced care and caution provided to individuals with multimorbidity, resulting in a higher perceived level of satisfaction than those without comorbid conditions. The level of satisfaction is also often recognised as a proxy indicator for the appropriateness, efficacy, quality, and feasibility of care with the potential to guide policymakers and guidelines development (Rossettini et al. 2018). However, no study has investigated whether metabolically unhealthy individuals receive different treatments for OA and whether metabolic conditions can impact healthcare satisfaction with OA management.

Thus, this study explored the association (i) between metabolic conditions and OA treatment utilisation and between metabolic conditions and satisfaction with OA care, which included (ii) satisfaction with received OA treatments and (iii) satisfaction with information about OA management from healthcare professionals.

## 2 Methods

### 2.1 Study Design and Data Source

This is a secondary analysis of a cross‐sectional web‐based survey study conducted in Italy, Sweden, and Russia between December 2021 and June 2022, originally designed to explore cross‐country OA management differences in adults with self‐reported knee or hip OA (Battista et al. 2024).

These three countries were chosen as representatives of three European regions: Mediterranean/Southern Europe (Italy), Northern Europe (Sweden), and Eastern Europe (Russia). In Italy, ethical approval was obtained from the Ethics Committee for University Research (CERA: Comitato Etico per la Ricerca di Ateneo), University of Genova (approval date: 15/06/2020; CERA2020.07). In Sweden and Russia, formal ethical approvals are not required for anonymous web‐based surveys that do not collect personally identifiable data. The research was conducted in respect of the Declaration of Helsinki (World Medical Association 2024). The survey was designed according to the ‘International Handbook of Survey Methodology’ (Dillman, de Leeuw, and Hox 2008) and reported following the ‘Strengthening the Reporting of Observational Studies in Epidemiology’ (STROBE) recommendations (Vandenbroucke et al. 2007). More detailed information about the survey can be found in Supporting Information S1 and in the original paper (Battista et al. 2024).

The survey was divided into five sections: (I) demographic and clinical data; (II) level of knowledge about different treatments for OA management; (III) treatments performed and suggested; (IV) expectations, beliefs and perceived barriers to OA management; (V) level of satisfaction with OA management.

Data for our study were retrieved from ‘Section 1’, where people were asked for information about age, years lived with OA, gender, education, comorbidities, pain, disability and country where OA management was received, ‘Section 3’, and ‘Section 5’. In particular, for comorbidities, participants were asked, ‘Are you having any of the following pathologies?’ providing five predefined answers (i.e., High blood pressure, Cardiovascular diseases, Diabetes, Pulmonary disease, Psychiatric disorders) and an additional free text box. For the level of satisfaction with OA management, participants were asked to indicate an integer on a scale from 0 to 100 their levels of satisfaction (continuous measure), where 0 corresponds to ‘Not satisfied at all’ and 100 to ‘Fully satisfied’. The questions for the two outcomes were ‘Could you indicate your overall level of satisfaction with the treatment received for osteoarthritis?’ and ‘Could you indicate your overall level of satisfaction with the information received from the healthcare professionals for the treatment of osteoarthritis?’. Treatment utilisation was self‐reported via a multiple‐choice question listing recommended and non‐recommended treatments for OA, stating, ‘Indicate which of these treatments you have taken to manage osteoarthritis’.

Different dissemination strategies were adopted in the various countries. In Italy, the questionnaire was disseminated through patients' association categories (ANMAR—National Rheumatic Patient Association and AMAR Piemonte—Rheumatic Patient Association Piemonte), social media outlets, words‐of‐mouth among healthcare professionals, and ‘Livio Sciutto’ Foundation. The questionnaire was disseminated in Sweden through social media outlets and the Artrosportalen site. In Russia, the questionnaire was integrated into the Moscow branch of the Russian Rheumatological Association ‘Nadezhda’ newsletter.

### 2.2 Participants and Exposure

People aged 40 years or older with self‐reported knee or hip OA who completed the survey were eligible for this study (Zhang et al. 2010). To investigate the association between the presence of metabolic conditions (exposure) and (i) treatment utilisation, (ii) the perceived satisfaction with OA care, and (iii) the information they received about OA management (outcomes), we divided the population into two cohorts based on our exposure variable (binary variable ‐ yes/no): in one cohort we selected people with metabolic conditions and in the other people without metabolic conditions. We defined people as ‘exposed’ if they presented any of the following metabolic conditions: hypertension, type II diabetes, obesity, or dyslipidaemia. Information on the exposure was retrieved from Section 1 of the survey. The conditions entered in the free box were independently evaluated case by case by two researchers for possible metabolic condition classification, with a third researcher resolving any disputes. Obesity was defined as a Body Mass Index (BMI) ≥ 30, computed from self‐reported weight and height.

### 2.3 Statistical Analysis

The sample's characteristics were described by exposure (with or without metabolic conditions).

### 2.4 Treatment Utilisation

To study the association between having one or more metabolic condition(s) and treatment utilisation, we estimated the prevalence and the 95% confidence interval (CI) of individuals who utilised a specific treatment separately for individuals with and without metabolic conditions. We then compared the two groups, computing each treatment's prevalence ratio and 95% CI (prevalence OA and no metabolic conditions used as reference). To allow comparison between groups, the prevalences were adjusted for age, years lived with osteoarthritis, gender, most affected joint, educational attainment and country, using the direct standardisation technique (Fay and Feuer 1997). Values in the prevalence ratio above 1 suggest higher prevalence in people with metabolic conditions, while values between 0 and 1 suggest higher prevalence in people without.

### 2.5 Satisfaction With OA Management

We developed two hierarchical models (Weitkunat and Wildner 2002) to study the association between having one or more metabolic condition(s) and the individuals' satisfaction with OA management: (i) one with the perceived satisfaction with OA care and (ii) with the information they received about OA management. Both variables were handled as continuous variables (0 and 100). We used a mixed‐effect multiple linear regression with a forced entry method for the variables selected as confounders, with people nested within the country (i.e. Sweden, Italy, Russia) and fitted as a random effect. The confounding variables age (continuous), years lived with OA (continuous), gender (categorical: male, female, other), most affected joint (categorical: knee, hip, knee and hip), and educational attainment (categorical: primary education, secondary education, upper secondary education) were fitted as fixed effects. We used the Restricted Maximum Likelihood approach (REML) for the parameter estimation. The multicollinearity test reported a low correlation between variables in terms of the generalised variance inflation factor. Assumptions for the linear mixed effect model were not significantly violated. Associations between the presence of metabolic condition(s) (exposure) and satisfaction with the healthcare experience in OA care (outcome) were reported as the regression estimated betas (and their respective 95% CIs) representing the estimated difference in the mean satisfaction level for individuals with metabolic condition(s) compared to those without. In our model, positive beta suggests that, on average, individuals with metabolic condition(s) tend to report higher satisfaction levels. In comparison, a negative beta implies lower satisfaction levels for individuals with metabolic condition(s). These estimates were adjusted to reflect the influence of other variables in the model. We also report the intraclass correlation coefficient (ICC) and the *R* ^2^ to describe the model fit.

Given the differences in healthcare delivery for OA across different countries (Battista et al. 2024), which can influence patient satisfaction with the healthcare experience, we conducted a sensitivity analysis stratifying the model by country. Thus, we ran three multiple linear regressions for each country with variables fitted as fixed effects.

## 3 Results

### 3.1 Patients Characteristics

**FIGURE 1:**
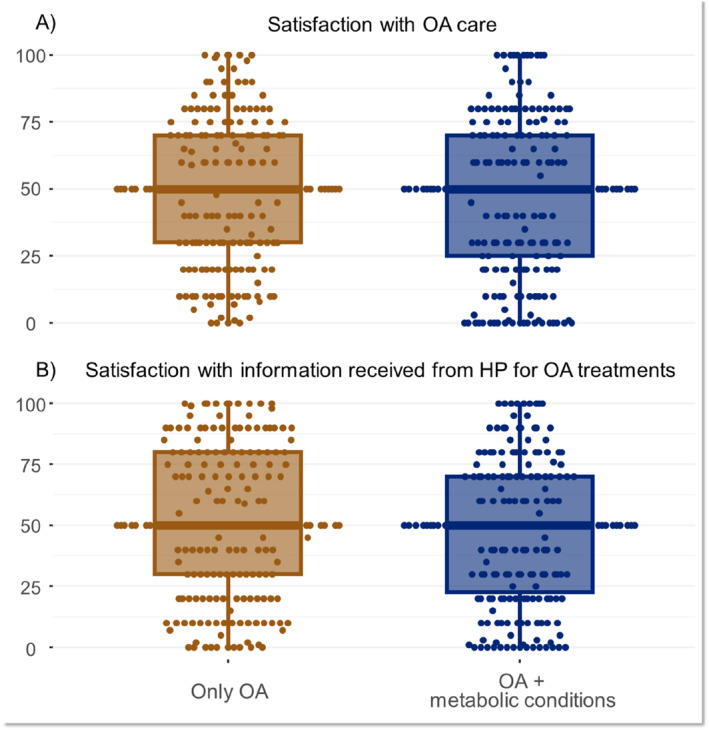
Box plot stratified by exposure (having at least one metabolic condition) for the distribution of (A) level of satisfaction with osteoarthritis (OA) care and (B) level of satisfaction with information received from healthcare professionals (HP) for OA treatments.

We collected surveys from 401 people with knee or hip OA and 40 years or older (Table 1). Sweden was the most represented country, with 48% (193) of participants, followed by Italy (28%, 111 participants) and Russia (24%, 97 participants). About half of the included individuals, 215 (54%), had at least one metabolic condition. Socio‐demographic characteristics between people with and without metabolic condition(s) were similar. Individuals with at least one metabolic condition were, on average, older (62 vs. 57 years old), had more often knee OA (82% vs. 62%) and had lower educational attainment (51% vs. 61% with an upper secondary education). Pain intensity and disability (both 0–100) were similar between groups. Boxplots of the actual values of satisfaction with OA care and satisfaction with information received from healthcare professionals regarding OA management are reported in Figure 1.

**TABLE 1:** Descriptive statistics.

	Individuals with OA and without metabolic conditions^a^ (*N* = 186)	Individuals with OA and metabolic conditions^a^ (*N* = 215)	Overall (*N* = 401)
Age			
Mean (SD)	57.3 (9.4)	61.8 (9.6)	59.7 (9.8)
Years of OA			
Mean (SD)	7.8 (7.6)	8.8 (7.3)	8.3 (7.5)
Gender			
Male	43 (23.1%)	44 (19.5%)	85 (21.2%)
Female	143 (76.9%)	171 (79.5%)	314 (78.3%)
Other	0 (0%)	2 (0.9%)	2 (0.5%)
Educational attainment			
Primary education	12 (6.5%)	31 (14.4%)	43 (10.7%)
Secondary education	61 (32.8%)	75 (34.9%)	136 (33.9%)
Upper secondary education	113 (60.8%)	109 (50.7%)	222 (55.4%)
Most affected joint			
Hip	71 (38.2%)	38 (17.7%)	109 (27.2%)
Knee	70 (37.6%)	105 (48.8%)	175 (43.6%)
Hip and knee	45 (24.2%)	72 (33.5%)	117 (29.2%)
Country			
Italy	56 (30.1%)	55 (25.6%)	111 (27.7%)
Sweden	90 (48.4%)	103 (47.9%)	193 (48.1%)
Russia	40 (21.5%)	57 (26.5%)	97 (24.2%)
Pain intensity (0–100)			
Mean (SD)	58.6 (22.0)	61.3 (22.4)	60.0 (22.2)
Disability intensity (0–100)			
Mean (SD)	62.8 (22.7)	67.0 (23.1)	65.0 (23.0)
Overall satisfaction with OA care (0–100)			
Mean (SD)	50.7 (30.2)	47.1 (28.5)	48.9 (29.3)
Overall satisfaction with information received about OA management (0–100)			
Mean (SD)	49.3 (26.6)	46.5 (28.5)	47.8 (27.6)
Treatment prevalence			
Antidepressants	14 (7.2%)	23 (11.1%)	37 (9.2%)
Corticosteroid injection	54 (27.8%)	78 (37.7%)	132 (32.9%)
Education programs	186 (95.9%)	195 (94.2%)	381 (95.0%)
Electric physical therapy	48 (24.7%)	50 (24.2%)	98 (24.4%)
Exercise	141 (72.7%)	157 (75.8%)	298 (74.3%)
Homoeopathy	8 (4.1%)	16 (7.7%)	24 (6.0%)
Hyaluronic acid injection	41 (21.1%)	40 (19.3%)	81 (20.2%)
Manual therapy	73 (37.6%)	63 (30.4%)	136 (33.9%)
Natural therapy	38 (19.6%)	34 (16.4%)	72 (18.0%)
NSAIDs	108 (55.7%)	114 (55.1%)	222 (55.4%)
Opioids	18 (9.3%)	34 (16.4%)	52 (13.0%)
Orthosis	87 (44.8%)	91 (44.0%)	178 (44.4%)
Other physical therapies	105 (54.1%)	128 (61.8%)	233 (58.1%)
Acetaminophen (paracetamol)	60 (30.9%)	66 (31.9%)	126 (31.4%)
Surgery	33 (17.0%)	32 (15.5%)	65 (16.2%)
Weight management	158 (81.4%)	157 (75.8%)	315 (78.6%)

Abbreviations: OA, osteoarthritis; SD, standard deviation.

^a^ individuals are considered with metabolic conditions if they reported at least one of the following conditions: hypertension, type II diabetes, obesity, or dyslipidaemia.

### 3.2 Treatment Utilisation

**FIGURE 2:**
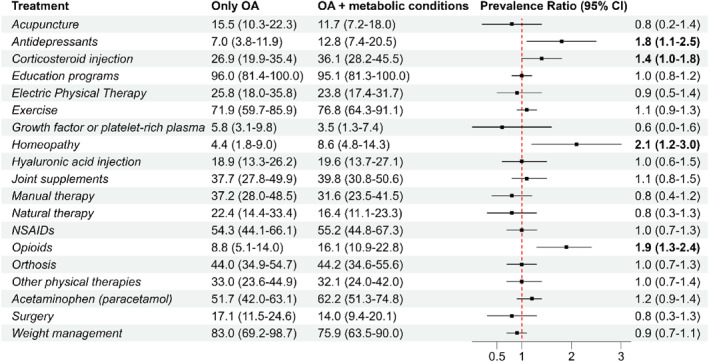
Prevalence of treatment utilisation (in %) by exposure (having at least one metabolic condition) and prevalence ratio. Standardised prevalences are expressed as percentages with 95% confidence interval. Prevalence ratios between 0 and 1 suggest higher prevalence in individuals without metabolic conditions, whereas prevalence ratios bigger than 1 suggest higher prevalence in individuals with metabolic conditions. Prevalences and prevalence ratios are adjusted for age, year lived with OA, gender, country, and most affected joint and education attainment.

Previous use of opioids (standardised prevalence ratio [95% CI] of 1.9 [1.3–2.4]), antidepressants (1.8 [1.1–2.5]), corticosteroid injections (1.4 [1.0–1.8]), and homoeopathic products (2.1 [1.2–3.0]) was higher in individuals with metabolic conditions compared to those without these conditions when direct standardised prevalence ratios were computed (Figure 2). The other treatment modalities showed similar prevalence across groups, with education programs (standardised prevalence of 96% vs. 95% in individuals with OA and OA + metabolic conditions, respectively), weight management (83% vs. 76%), and exercise (72% vs. 77) being the most common.

### 3.3 Satisfaction

There was no association between metabolic condition(s) and satisfaction with OA care (adjusted difference of −3.1 [95% CI: −8.5; 2.4]) or satisfaction with information received from healthcare professionals regarding OA management (−4.0 [95% CI: −9.7; 1.7]). The ICC suggested a relatively low level of agreement in the perceived satisfaction among the individuals within the same country (0.13 for satisfaction in OA care and 0.12 for satisfaction in the information received). The *R* ^2^ value of our models showed that all the factors that we selected and used in the models explained approximately 11.6% of the total variance in the satisfaction levels with OA care and 13.5% in the satisfaction with the information received. More details about the regression results of the main analysis are reported in Table 2. The stratified analysis between countries confirmed the main analysis results, which did not detect an association between metabolic condition(s) and satisfaction with OA care or information received from healthcare professionals regarding OA management. More details about regression results of the sensitivity analysis are reported in Supporting Information S1: Table S1.

**TABLE 2:** Regression results for the main analysis.

Regression parameters (estimate [95% CI])	Satisfaction with OA care	Satisfaction with information received for OA management
(Intercept)	33.1 (10.5–55.8)	39.2 (15.8–62.7)
Metabolic conditions^a^	−3.1 (−8.5–2.4)	−4.0 (−9.7–1.7)
Education		
Primary	(Reference category)	
Secondary	1.7 (−7.6–11.0)	1.3 (−8.4–11.0)
Upper secondary	1.6 (−7.6–10.9)	0.2 (−9.5–10.0)
Gender		
Man	(Reference category)	
Woman	−4.3 (−11.2–2.6)	−9.2 (−16.5–−1.9)
Other	−5.3 (−42.3–31.7)	−17.0 (−55.9–21.9)
Joint		
Hip	(Reference category)	
Knee	3.1 (−3.4–9.6)	−0.1 (−7.0–6.7)
Hip and knee	1.3 (−6.0–8.4)	−1.2 (−8.8–6.3)
Age	0.3 (−0.0–0.6)	0.3 (−0.0–0.6)
Years of OA	0.1 (−0.2–0.5)	0.2 (−0.2–0.5)
Model statistics		
Adjusted ICC	0.13	0.12
Random intercept estimates for countries		
Italy	43.4	49.8
Sweden	32.5	37.4
Russia	23.6	30.5

Abbreviations: CI, confidence interval; ICC, intraclass correlation coefficient; OA, osteoarthritis.

^a^ individuals are considered with metabolic conditions if they reported at least one of the following conditions: hypertension, type II diabetes, obesity, or dyslipidaemia.

## 4 Discussion

This study explored the association between metabolic conditions, treatment utilisation for OA, and perceived satisfaction with OA management across three European countries. Our findings suggest that individuals with and without metabolic conditions have a similar prevalence in the utilisation of first‐line interventions for OA, such as exercise, education, and weight management. However, individuals with metabolic conditions tended to rely more on second‐line optional interventions, including opioids, corticosteroid injections, and antidepressants, as well as non‐recommended treatments such as homoeopathic products. Despite these treatment utilisation differences, they reported similar satisfaction with OA management.

Second‐line treatments and medications can be expected to be more commonly used by people with multimorbidity, as first‐line treatments tend to have reduced effectiveness on symptoms compared to metabolically healthy individuals (Andrea et al. 2024; Pihl et al. 2021), and some national and international clinical guidelines conditionally recommend them (Bannuru et al. 2019; Kolasinski et al. 2020; Moseng et al. 2024; National Clinical Guidelines Centre 2022). However, since not all individuals reported receiving first‐line treatments, more efforts might be needed to engage a larger number of individuals with OA and metabolic conditions in treatments that promote lifestyle changes, as they are the ones who could benefit the most. Moreover, developing more effective and personalised first‐line intervention programmes for individuals with OA and metabolic conditions involving specialists in OA and metabolic health management could reduce the use of second‐line treatments, reducing the adverse events associated with pharmacological treatments.

Further consideration must be given to the potential side effects and risk‐benefits of opioids, antidepressants, and corticosteroids, as our study indicates that these are more prevalent in individuals with OA and metabolic conditions. Despite opioids being thought to have a safer cardiovascular profile than NSAIDs and to have low effectiveness for OA pain, there is strong evidence indicating an increased risk of side effects, withdrawal symptoms, and other adverse events that result in serious health consequences when used for OA compared to placebo treatments (Costa et al. 2014; Megale et al. 2018). The low risk‐benefit has led the medical and scientific community to question its actual usefulness, prompting some international clinical guidelines to advise against the use of oral or transdermal opioids for OA pain management (Bannuru et al. 2019). Antidepressants tend to be conditionally recommended by guidelines, although they are not as effective (Ferreira et al. 2023; Stannard and Wilkinson 2023). Moreover, a systematic review with meta‐analysis on the adverse effects of antidepressants for chronic pain revealed an increased risk of side effects against placebo, although, compared with opioids, they more rarely lead to serious adverse events, particularly in the case of low dosages (Riediger et al. 2017). However, some of the most common adverse events, such as dizziness, palpitations, and drowsiness, could increase the already significant barriers to physical activity in individuals with OA and metabolic conditions (Armstrong, Colberg, and Sigal 2023; Baillot et al. 2021; McIntosh, Hunter, and Royce 2016; Metsios et al. 2023). Long‐term corticosteroid injections also have potential complications, especially when used repeatedly over a short time, such as tissue atrophy, calcification, sepsis, acceleration of cartilage damage, vascular necrosis, and haematoma (Kompel et al. 2019; Stone, Malanga, and Capella 2021). Therefore, opioids, antidepressants, and corticosteroids for OA may not always be appropriate for individuals with multimorbidity and their prescription should be discouraged, even if first‐line treatments fail to provide adequate relief or if the individual's risk profile makes NSAIDs unsuitable (Bannuru et al. 2019).

The similar level of satisfaction with the management of OA between individuals with and without metabolic conditions can be explained by the trade‐off between the positive effect of increased attention and more comprehensive care by health professionals for individuals with multimorbidity and the negative effect of increased health care burden for individuals associated with multimorbidity. This is highlighted by the similar prevalence of first‐line treatments such as exercise, education and weight management, but higher prevalence of second‐line treatments, non‐recommended treatments and even alternative medicine treatments in metabolically unhealthy individuals. Higashi et al. (Higashi et al. 2007) offered a similar explanation for the association between better quality of care and increased coexisting long‐term conditions, interpreting their results with the increased attention received by people with multimorbidity. This phenomenon can result in dispensing more treatments that may increase satisfaction with care, as may have happened in our study.

Another explanation for the similar level of satisfaction arises from the experiences of individuals with multimorbidity who may undergo shifts in their self‐assessment of quality of life due to long‐term conditions (Ilie et al. 2019). Response shift refers to changes in an individual's self‐assessment of a specific construct due to (a) alterations in the internal standards used for measurement (referred to as scale recalibration in psychometric contexts); (b) shifts in values, affecting the importance attributed to different aspects of the target construct; or (c) reinterpretation or reconceptualisation of the target construct itself. In our context, individuals with multimorbidity may have adjusted their evaluation of satisfaction with care received due to changes in internal standards, values, or a deeper understanding of the quality of life and expectations for future health (Vanier et al. 2021).

Notably, we also observed great variability in the perceived satisfaction between countries (43.4/49.8 in Italy, 32.5/37.4 in Sweden and 23.6/30.5 in Russia for satisfaction with OA care/information received) and between people within each country (a low ICC of 0.13/0.12 in the main analysis for satisfaction with OA care/information received). This result suggests that apart from substantial variability between countries, which may represent differences in healthcare systems, a significant portion of the variability could be attributed to factors within each country, such as geographic location (urban vs. rural, rich vs. poor) and individual healthcare providers (Batbaatar et al. 2017; Rossettini et al. 2018).

Some limitations of this study need to be acknowledged. Because of the cross‐sectional design, we cannot establish causality between metabolic conditions, treatment utilisation, and different levels of satisfaction in OA management. We also did not know at what point of the treatment path people were included in the study. Moreover, since we used an online voluntary survey without a computed response rate, a sampling bias and a classification bias may have been introduced. The former may have led us to an unrepresentative sample of the population due to a substantial difference between responders and non‐responders. The latter may have led us to select people without OA or to misclassify those exposed or unexposed to metabolic conditions due to the self‐reporting nature of the data collected. A second limitation comes from the outcomes used for the analyses. For treatment utilisation, self‐reported data may be inaccurate due to recall bias. Additionally, we did not collect information on whether and how treatments such as exercise were adapted to the presence of metabolic comorbidities. Moreover, assessing satisfaction with OA management using a single score may be insufficient as satisfaction is a multidimensional construct. People could anchor their satisfaction, for instance, to the outcome of an intervention, the appropriateness of treatments, the pleasantness of healthcare staff, or the cleaning of facilities. Nonetheless, single overall scores in questionnaires are widely used both in the research and in the evaluation of health systems and are used to improve the healthcare experience (Friedel et al. 2023).

On the other hand, the generalisability of our results benefited from the cross‐national design incorporating data from three European countries that are representative of three major European areas. Furthermore, the big difference in variance that we saw in the level of satisfaction between countries and within countries provided interesting insights for further study to improve our understanding of the whole OA management experience in people with metabolic conditions. Further longitudinal data on the frequency, dosages and adaptation of treatments for OA, including first‐line interventions, in people with metabolic conditions may provide further insight into the impact of metabolic health on OA management.

## 5 Conclusions

Individuals with OA and metabolic conditions received opioids, corticosteroid injections, antidepressants, and homoeopathy more often than metabolically healthy OA individuals, while first‐line interventions were provided similarly in both groups. This may be due to the reduced effectiveness of first‐line treatments, leading to the search for alternative solutions. Nonetheless, satisfaction levels were similar between groups, likely due to the balance between increased attention and comprehensive care, and the negative impact of a higher healthcare burden. Greater efforts may be needed to engage individuals with OA and metabolic conditions in lifestyle interventions whilst reducing the reliance on treatments with a worse risk‐benefit ratio and developing more effective personalised programmes focused on exercise, education, and weight management.

## Author Contributions

All authors significantly contributed to the study's conception, design, data analysis, manuscript writing, revision, and approval for publication. They are all accountable for ensuring the work's accuracy and integrity and addressing any concerns related to it.

## Ethics

In Italy, ethical approval was obtained from the Ethics Committee for University Research (CERA: Comitato Etico per la Ricerca di Ateneo), University of Genova (approval date: 15/06/2020; CERA2020.07). In Sweden and Russia, no Ethics Committee approvals were necessary. This study was reported according to the ‘Strengthening the Reporting of Observational Studies in Epidemiology’ (STROBE) recommendations (Vandenbroucke et al. 2007).

## Conflicts of Interest

The authors declare no conflicts of interest.

## Supporting information

Supporting Information S1

## Data Availability

The dataset used and analysed during the current study is available from the corresponding author on reasonable request.
