# Supplementary material for: Treatment Utilisation and Satisfaction With Management in Individuals With Osteoarthritis and Metabolic Multimorbidity: A Cross‐Sectional Multi‐Country Study
Source: Musculoskeletal Care. 2025 Jan 23;23(1):e70058. doi: 10.1002/msc.70058 (PMC11757013; doi:10.1002/msc.70058)
Supplement: Supplementary file 1 — Supporting Information S1 [file MSC-23-e70058-s001.docx]

# Supplementary Materials

Supplementary Table 1. Regression results for the sensitivity analysis

|  | **Italy** | | **Sweden** | | **Russia** | |
| --- | --- | --- | --- | --- | --- | --- |
| **Regression parameters (estimate [95% CI])** | **Satisfaction with OA care** | **Satisfaction with information received for OA management** | **Satisfaction with OA care** | **Satisfaction with information received for OA management** | **Satisfaction with OA care** | **Satisfaction with information received for OA management** |
| (Intercept) | 57.9 (24.7 - 91.1) | 58.7 (25.7 - 91.8) | 27.7 (-5.9 - 61.3) | 36.7 (2.2 - 71.3) | -15.3 (-61.3 - 30.7) | 4.2 (-50.6 - 58.9) |
| **Metabolic conditions*** | -6.8 (-17.3 - 3.7) | -6.9 (-17.3 - 3.5) | -1.6 (-10.1 - 6.8) | -4.1 (-12.7 - 4.6) | -4.8 (-15.0 - 5.4) | -2.2 (-14.4 - 9.9) |
| **Education** |  |  |  |  |  |  |
| Primary |  | |  |  |  |  |
| Secondary | -5.9 (-15.6 - 3.9) | -7.5 (-17.3 - 2.2) | -3.4 (-14.9 - 8.0) | -8.6 (-20.4 - 3.1) | 16.6 (-7.7 - 40.9) | -3.3 (-32.2 - 25.6) |
| Upper Secondary | -9.7 (-61.4 - 41.9) | -30.8 (-82.2 - 20.5) | -1.4 (-58.4 - 55.6) | -4.4 (-63.0 - 54.2) | -6.7 (-29.1 - 15.7) | 0.0 (-26.7 - 26.7) |
| **Gender** |  |  |  |  |  |  |
| Man |  | |  |  |  |  |
| Woman | -2.5 (-15.4 - 10.5) | -1.0 (-13.9 - 11.9) | 12.4 (-6.0 - 30.8) | 9.7 (-9.2 - 28.6) | -1.9 (-23.3 - 19.4) | 4.2 (-21.3 - 29.6) |
| Other | -4.8 (-18.5 - 9.0) | -10.3 (-24.0 - 3.3) | 12.2 (-5.6 - 30.1) | 10.3 (-8.0 - 28.7) | 0.6 (0.1 - 1.1) | 0.5 (-0.2 - 1.1) |
| **Joint** |  |  |  |  |  |  |
| Hip |  |  |  |  |  |  |
| Knee | -2.7 (-14.1 - 8.8) | -8.8 (-20 - 2.5) | 7.2 (-3.1 - 17.5) | 3.8 (-6.8 - 14.4) | 4 (-9.5 - 17.4) | 5.8 (-10.2 - 21.8) |
| Hip and knee | -3.9 (-17.9 - 10) | -6.3 (-20 - 7.5) | 3.4 (-8.4 - 15.3) | -0.4 (-12.6 - 11.7) | 3.7 (-9.1 - 16.5) | 3.5 (-11.7 - 18.8) |
| **Age** | 0.2 (-0.3 - 0.7) | 0.3 (-0.3 - 0.8) | 0.2 (-0.3 - 0.7) | 0.2 (-0.3 - 0.7) | 1.1 (0.3 - 1.8) | 1.1 (0.2 - 2.0) |
| **Years of OA** | -0.2 (-1.2 - 0.8) | 0.0 (-1.0 - 1.0) | 0.0 (-0.5 - 0.4) | 0.0 (-0.5 - 0.5) | -0.2 (-1.2 - 0.8) | -0.2 (-1.2 - 0.8) |
| **Model statistics** |  |  |  |  |  |  |
| **R^2^** | 0.04 | 0.09 | 0.02 | 0.03 | 0.1 | 0.14 |
| * individuals are considered with metabolic conditions if they reported at least one of the following conditions: hypertension, type II diabetes, obesity, dyslipidaemia.  Abbreviation: osteoarthritis (OA); coefficient of determination (R²); confidence interval (CI) | | | | | | |

Supplementary File 1. English version of the survey

| **Did you receive a diagnosis of either hip osteoarthritis or knee osteoarthritis or hip and knee osteoarthritis?** | | Yes/No |
| --- | --- | --- |
| **Section 1 – Descriptive Analysis** | | |
| Age (insert number): | |  |
| To which gender do you most identify with? | | Male |
|  |  | Female |
|  |  | Other: |
|  |  | Prefer not to answer: |
| If you answered "Other" to the question above, please write down which gender you most identify with: | |  |
| In which country are you attending your cure for osteoarthritis? | | Insert nationality: |
| Are you living alone? | | Yes |
|  |  | No |
| Job/Profession: | | Sedentary |
|  |  | Dynamic |
|  |  | Heavy |
|  |  | Retired |
|  |  | Unemployed |
| Educational level: | | Primary |
|  |  | Lower Secondary |
|  |  | Higher Secondary |
|  |  | University |
| Weight: | |  |
| Height: | |  |
| Do you practise any sports or physical activity on a regular basis? | | Yes/no |
| If yes, how many time per week? | | 1-2 |
|  |  | 3-5 |
|  |  | 6-7 |
|  |  | >7 |
| Joint(s) affected by Osteoarthritis: | | Hip |
|  |  | Knee |
|  |  | Hip and Knee |
| How long have you suffered from Osteoarthritis (Please report years e.g. 4): | |  |
| Are you having any of the following pathologies: | | High blood pressure |
|  |  | Cardiovascular diseases |
|  |  | Diabetes |
|  |  | Pulmonary disease |
|  |  | Psychiatric disorders (Major depression, generalised anxiety disorders etc.) |
|  |  | Other: |
| If you answered "Other" to the previous answer please write down which pathology/ies: | |  |
| How would you quantify the most frequent pain caused by osteoarthritis on a scale from 0 to 100, where 0 corresponds to "No pain" and 100 a "Maximum bearable pain"? | |  |
| How would you quantify to what extent the symptoms of osteoarthritis have affected your joint mobility on a scale from 0 to 100, where 0 corresponds to "Not affected" and 100 "Seriously affected"? | |  |
| Are you on a waiting list for total joint replacement? | | Yes |
|  |  | No |
| Which professional(s) did you turn to for your problem? (You can select more than one option) | | Physician |
|  |  | Physiotherapist |
|  |  | Sport Scientist/Personal trainer/Coach |
|  |  | Chiropractor |
|  |  | Osteopath |
|  |  | Other: |
| If you answered "Other" do the previous answer please specify which professionals: | |  |
| Have you ever followed a specific diet protocol for OA (e.g. paleodiet, intermittent fasting etc.) | | Yes |
|  |  | No |
| If you answered "yes" to the previous question, please specific which diet protocol did you follow: | |  |
| **Section 2 – Level of Knowledge** | | |
| Please, indicate how you consider the following treatments (Recommended: compulsory treatment) (Optional: optional treatment) (Not recommended: treatment not recommended or to avoid) | | |
| Surgery (Total Joint Replacement) | Recommended | |
|  | Optional | |
|  | Not recommended | |
|  | I do not know | |
| Electrical Physical Therapy (e.g. TENS) | Recommended | |
|  | Optional | |
|  | Not recommended | |
|  | I do not know | |
| Other physical therapies (Laser, Ultrasound, electromagnetic field etc.) | Recommended | |
|  | Optional | |
|  | Not recommended | |
|  | I do not know | |
| Acupuncture | Recommended | |
|  | Optional | |
|  | Not recommended | |
|  | I do not know | |
| Topical anti-inflammatory drugs (e.g. cream) | Recommended | |
|  | Optional | |
|  | Not recommended | |
|  | I do not know | |
| Oral anti-inflammatory drugs | Recommended | |
|  | Optional | |
|  | Not recommended | |
|  | I do not know | |
| Acetaminophen (e.g. paracetamol) | Recommended | |
|  | Optional | |
|  | Not recommended | |
|  | I do not know | |
| Opioid drugs | Recommended | |
|  | Optional | |
|  | Not recommended | |
|  | I do not know | |
| Antidepressant drugs | Recommended | |
|  | Optional | |
|  | Not recommended | |
|  | I do not know | |
| Hyaluronic acid injections | Recommended | |
|  | Optional | |
|  | Not recommended | |
|  | I do not know | |
| Glucocorticoid steroid injections | Recommended | |
|  | Optional | |
|  | Not recommended | |
|  | I do not know | |
| Growth factor injections and/or platelet-rich plasma | Recommended | |
|  | Optional | |
|  | Not recommended | |
|  | I do not know | |
| Joint supplements (glucosamine, chondroitin) | Recommended | |
|  | Optional | |
|  | Not recommended | |
|  | I do not know | |
| Homeopathic therapies | Recommended | |
|  | Optional | |
|  | Not recommended | |
|  | I do not know | |
| Natural Therapy (Topic Herbal therapies, natural supplements e.g. devil's claw, clay compress) | Recommended | |
|  | Optional | |
|  | Not recommended | |
|  | I do not know | |
| Weight loss for overweight or obese people | Recommended | |
|  | Optional | |
|  | Not recommended | |
|  | I do not know | |
| Maintain healthy weight for normal weight range people | Recommended | |
|  | Optional | |
|  | Not recommended | |
|  | I do not know | |
| General physical activity (yoga, tai-chi, pilates, nordic walking) | Recommended | |
|  | Optional | |
|  | Not recommended | |
|  | I do not know | |
| Specific Physical exercise (combination of aerobic exercise and strengthening) | Recommended | |
|  | Optional | |
|  | Not recommended | |
|  | I do not know | |
| Balance training (proprioception exercises) | Recommended | |
|  | Optional | |
|  | Not recommended | |
|  | I do not know | |
| Manual Therapy (massage, mobilisations, manipulations) | Recommended | |
|  | Optional | |
|  | Not recommended | |
|  | I do not know | |
| Orthesis or walking aids (comfortable footwear, braces or aids) | Recommended | |
|  | Optional | |
|  | Not recommended | |
|  | I do not know | |
| Education programmes (e.g. goal setting, skill building, education about exercise etc.) | Recommended | |
|  | Optional | |
|  | Not recommended | |
|  | I do not know | |
| Sleep-management strategies (mindfulness, drugs, psychologist etc.) | Recommended | |
|  | Optional | |
|  | Not recommended | |
|  | I do not know | |
| Stress-management strategies (mindfulness, drugs, psychologist etc.) | Recommended | |
|  | Optional | |
|  | Not recommended | |
|  | I do not know | |
| **Section 3 – Treatments Performed and Suggested** | | |
| Indicate which treatment(s) was/were ever recommended to you by a **health professional** (e.g. medical doctor, physiotherapists, nutritionist): | | |
| (Here we have the same treatments mentioned above in part 3). | | |
| Indicate which of these treatments you have actually taken to manage osteoarthritis: | | |
| (Here we have the same treatments mentioned above in part 3). | | |
| **Section 4 – Expectations, Beliefs and Perceived Barriers to OA Management** | | |
| **Indicate to what extent you agree or disagree with the following statements regarding OSTEOARTHRITIS (Strongly disagree/Disagree/Neither Disagree or Agree/Agree/Strongly Agree)** | | |
| I perceive the OA treatments as uncertain (i.e. with no precise care process) | Strongly disagree | |
|  | Partially Disagree | |
|  | Neither disagree nor agree | |
|  | Partially Agree | |
|  | Strongly Agree | |
| I search the internet for answers to my disease-related concerns and doubts | Strongly disagree | |
|  | Partially Disagree | |
|  | Neither disagree nor agree | |
|  | Partially Agree | |
|  | Strongly Agree | |
| The health professionals I met did not address my doubts regarding the treatment of my disease | Strongly disagree | |
|  | Partially Disagree | |
|  | Neither disagree nor agree | |
|  | Partially Agree | |
|  | Strongly Agree | |
| I perceive OA as a disease of the ageing process | Strongly disagree | |
|  | Partially Disagree | |
|  | Neither disagree nor agree | |
|  | Partially Agree | |
|  | Strongly Agree | |
| Sooner or later I will have to undergo surgical intervention (total joint replacement) to solve my problem | Strongly disagree | |
|  | Partially Disagree | |
|  | Neither disagree nor agree | |
|  | Partially Agree | |
|  | Strongly Agree | |
| **Indicate to what extent you agree or disagree with the following statements regarding non-surgical treatments (Strongly disagree/Disagree/Neither Disagree or Agree/Agree/Strongly Agree)** | | |
| Exercise should be avoided in OA management | Strongly disagree | |
|  | Partially Disagree | |
|  | Neither disagree nor agree | |
|  | Partially Agree | |
|  | Strongly Agree | |
| Exercise is effective for everyone, regardless of pain severity | Strongly disagree | |
|  | Partially Disagree | |
|  | Neither disagree nor agree | |
|  | Partially Agree | |
|  | Strongly Agree | |
| Physical exercise should only be undertaken after the prescription of drug treatments to control pain | Strongly disagree | |
|  | Partially Disagree | |
|  | Neither disagree nor agree | |
|  | Partially Agree | |
|  | Strongly Agree | |
| Radiographic findings are necessary before starting physical exercise | Strongly disagree | |
|  | Partially Disagree | |
|  | Neither disagree nor agree | |
|  | Partially Agree | |
|  | Strongly Agree | |
| Rest from physical exercise is needed in case of severe osteoarthritis | Strongly disagree | |
|  | Partially Disagree | |
|  | Neither disagree nor agree | |
|  | Partially Agree | |
|  | Strongly Agree | |
| Low-impact physical exercise (es. pilates, swimming) is preferrable to high-impact exercise (strengthening, walking, running etc.) | Strongly disagree | |
|  | Partially Disagree | |
|  | Neither disagree nor agree | |
|  | Partially Agree | |
|  | Strongly Agree | |
| Physical exercise is useful also to prevent other health problems related to OA (e.g. diabetes, heart disease etc.) | Strongly disagree | |
|  | Partially Disagree | |
|  | Neither disagree nor agree | |
|  | Partially Agree | |
|  | Strongly Agree | |
| The main role of physiotherapy before surgery is to reduce pain while waiting for it | Strongly disagree | |
|  | Partially Disagree | |
|  | Neither disagree nor agree | |
|  | Partially Agree | |
|  | Strongly Agree | |
| The main role of physiotherapy in OA management is after surgery | Strongly disagree | |
|  | Partially Disagree | |
|  | Neither disagree nor agree | |
|  | Partially Agree | |
|  | Strongly Agree | |
| Weight loss (when overweight or obese) is important for reducing the load on the joint in OA | Strongly disagree | |
|  | Partially Disagree | |
|  | Neither disagree nor agree | |
|  | Partially Agree | |
|  | Strongly Agree | |
| Weight loss (when overweight or obese) is important for reducing body inflammation in OA | Strongly disagree | |
|  | Partially Disagree | |
|  | Neither disagree nor agree | |
|  | Partially Agree | |
|  | Strongly Agree | |
| **Indicate to what extent these elements represent a barrier, for you, to doing exercise? (Strongly disagree/Disagree/Neither Disagree or Agree/Agree/Strongly Agree)** | | |
| Cost | Strongly disagree | |
|  | Partially Disagree | |
|  | Neither disagree nor agree | |
|  | Partially Agree | |
|  | Strongly Agree | |
| This treatment is not available in the area I live | Strongly disagree | |
|  | Partially Disagree | |
|  | Neither disagree nor agree | |
|  | Partially Agree | |
|  | Strongly Agree | |
| Lack of time | Strongly disagree | |
|  | Partially Disagree | |
|  | Neither disagree nor agree | |
|  | Partially Agree | |
|  | Strongly Agree | |
| Unwillingness | Strongly disagree | |
|  | Partially Disagree | |
|  | Neither disagree nor agree | |
|  | Partially Agree | |
|  | Strongly Agree | |
| It is not covered by my public insurance system/public health system | Strongly disagree | |
|  | Partially Disagree | |
|  | Neither disagree nor agree | |
|  | Partially Agree | |
|  | Strongly Agree | |
| I do not think it is useful for my problem | Strongly disagree | |
|  | Partially Disagree | |
|  | Neither disagree nor agree | |
|  | Partially Agree | |
|  | Strongly Agree | |
| No one has ever suggested me to do so | Strongly disagree | |
|  | Partially Disagree | |
|  | Neither disagree nor agree | |
|  | Partially Agree | |
|  | Strongly Agree | |
| Other (Please insert any other reason you may have): |  | |
| **Indicate to what extent these elements represent a barrier, for you, to weighting loss? (Strongly disagree/Disagree/Neither Disagree or Agree/Agree/Strongly Agree)** | | |
| Cost | Strongly disagree | |
|  | Partially Disagree | |
|  | Neither disagree nor agree | |
|  | Partially Agree | |
|  | Strongly Agree | |
| This treatment is not available in the area I live | Strongly disagree | |
|  | Partially Disagree | |
|  | Neither disagree nor agree | |
|  | Partially Agree | |
|  | Strongly Agree | |
| Lack of time | Strongly disagree | |
|  | Partially Disagree | |
|  | Neither disagree nor agree | |
|  | Partially Agree | |
|  | Strongly Agree | |
| Unwillingness | Strongly disagree | |
|  | Partially Disagree | |
|  | Neither disagree nor agree | |
|  | Partially Agree | |
|  | Strongly Agree | |
| It is not covered by my public insurance system/public health system | Strongly disagree | |
|  | Partially Disagree | |
|  | Neither disagree nor agree | |
|  | Partially Agree | |
|  | Strongly Agree | |
| I do not think it is useful for my problem | Strongly disagree | |
|  | Partially Disagree | |
|  | Neither disagree nor agree | |
|  | Partially Agree | |
|  | Strongly Agree | |
| No one has ever suggested me to do so | Strongly disagree | |
|  | Partially Disagree | |
|  | Neither disagree nor agree | |
|  | Partially Agree | |
|  | Strongly Agree | |
| Other (Please insert any other reason you may have): |  | |
| **Section 5 - Level of satisfaction** | | |
| Could you indicate your overall level of satisfaction with the treatment received for osteoarthritis on a scale from 0 to 100, where 0 corresponds to "Not satisfied at all" and 100 "Fully satisfied "? |  | |
| Could you indicate your overall level of satisfaction with the information received from the healthcare professionals for the treatment of osteoarthritis on a scale from 0 to 100, where 0 corresponds to "Not satisfied at all" and 100 "Fully satisfied "? |  | |
